# Hiccough and Its Treatment

**Published:** 1900-01-06

**Authors:** Carstairs Douglas

**Affiliations:** Professor of Medical Jurisprudence, Anderson's College Medical School, and Dispensary Physician, Samaritan Hospital for Women, Glasgow.


### HOSPITAL CLINICS AND MEDICAL PROGRESS.

### HICCOUGH AND ITS TREATMENT.

By CARSTAIRS DOUGLAS, M.D., B.Sc.Edin., Professor of Medical Jurisprudence, Anderson's College Medical School, and Dispensary Physician, Samaritan Hospital for Women, Glasgow.

HICCOUGH or Singultus is met with in various diseases, and may prove to be, by reason of its duration and severity, a symptom of the most refractory and distressing kind. It consists of a spasmodic involuntary contraction of the diaphragm, accompanied by a sharp inspiration, which from closure, complete or partial, of the glottis, gives rise to the well-known sound which is characteristic of the affection. The act is initiated by a stimulus, either reflex or direct, of the respiratory centre.

In the majority of cases in which it occurs it owes its origin to some gastric disturbance, and disappears, as a rule, in a few minutes, or at most hours, after the use of some simple expedient, such as taking a drink of water, holding the breath, &c., but it may persist for days, weeks, or months, and constitute a symptom of a most serious kind leading to great debility and even endangering life.

Afferent impulses giving rise to hiccough may reach the central nervous system through branches of the sympathetic arising from the solar and other plexuses, or through the ordinary nerves of sensation, or may take origin apparently in the central nervous mechanism without any peripheral source whatever. The efferent nerves concerned are the phrenics, and the laryngeal branches of the vagus.

Symes has classified this symptom under four heads:—

(1) Inflammatory: This is the variety met with in many abdominal affections (not purely gastric), such as hernia, appendicitis, or general peritonitis; while in typhoid fever hiccough is regarded by Poore as a sign of perforation. The impulses here travel along the visceral sympathetic nerves. I have seen this form occur in very early peritonitis after abdominal section, and if it persist for a few hours it should be regarded as a danger-signal. It often seems to occur most readily where the peritoneum in the vicinity of the diaphragm itself is affected, and may be present where the inflammation is so slight that the spasm causes no pain.

(2) Irritative: This group comprises all those cases where there is œsophageal, gastric, or intestinal disturbance apart from inflammation. This is the most common variety, and may be readily induced by undue haste in eating, the swallowing of very hot food, the ingestion of irritating substances into the stomach or bowel, and by the existence of flatus in these organs. This is the variety most amenable to treatment.

(3) Idiopathic: In these cases no clear peripheral cause can be made out; they occur in grave constitutional states, such as uræmia, gout, chronic Bright's disease, and diabetes. For example, a case has been recorded by Wagner where hiccough and slight œdema of the legs were the only symptoms in a case of chronic Bright's disease. This kind of hiccough may prove very intractable, depending, as it probably does, on a toxic condition of the blood, and may baffle all efforts of the physician to relieve it.

(4) Neurotic: Under this heading it is proposed to gather together cases of cerebral tumour, epilepsy, hysteria, and other affections of a nervous nature. It seems to me, however, that it would be better to designate this variety as "nervous," subdividing it into organic and functional if desired. The nervous variety, especially if functional, may be most obstinate. Dr. Wilson, of Florence, published notes of a case in 1895, where a woman with hysterical stigmata (ovarian, mammary, and spinal tenderness, &c.) suffered from hiccough for seven months.

While hiccough, as a rule, subsides readily under simple suitable treatment, it may last for many days (perhaps not absolutely continuous), and may greatly exhaust the patient. Sleep is interfered with, nutrition becomes impaired, and the heart's action may be rendered rapid, weak, and irregular. Some patients suffer much less in general health than others, but there may be great loss of flesh—a case having been reported in the *Boston Medical and Surgical Journal* for 1894 where the patient lost 70 lbs. in three or four months and finally succumbed. A case of similar duration was reported by Dr. Harrison, of Croydon, in 1895, but there was little loss of flesh, and the patient recovered completely. In these lengthy attacks there is usually some intermission, either of a few hours each day, or of some days each week. Were this not so, fatal results would be more common. I had, some months ago, a patient who had practically uninterrupted obstinate hiccough for five and a half days just after the crisis of a mild attack of pneumonia, and the effect on the heart and general state was very alarming. It yielded at length to sodium bromide.

When we come to consider treatment we find that this, in the main, is directed towards the nervous system, and just as hiccough is often induced by purely nervous conditions, so some sharp slight shock, physical or mental, will often serve to cause its disappearance. Holding the breath, which rests the diaphragm, or sipping cold water, which relieves gastric excitability, are simple and very frequently efficacious modes of relief. Should these fail, the use of a little ordinary snuff will often put an end to this annoying symptom. Should it persist longer, a warm poultice or a sinapism applied to the epigastrium may have the desired effect, and at the same time ice may be sucked. But if the attack be a really severe one, we may have to try many remedies, some of them of a potent kind, before relief is obtained. Most drugs employed are those of the nature of nerve sedatives or anti-spasmodics. Among those that have been found useful are morphine ($\frac{1}{4}$gr. hypodermically), belladonna, the extract in pill ($\frac{1}{4}$ to $\frac{1}{2}$ gr.) or the tincture (5 to 15♏); the bromides in 20 to 30 gr. doses with or without chloral hydrate gr. 15, musk 2 to 5 grs., pilocarpine gr. $\frac{1}{10}$ hypodermically, or tincture of jaborandi 30 to 60♏ menthol gr. $\frac{1}{2}$ to gr. 1 with or without sodium bicarbonate, carbolic acid 1 gr. in pill, valerianate of zinc or sulphate of quinine 2 grs. in pill, and tincture of cannabis indica 10 to 15 ♏. Inhalation of chloroform, of nitrite of amyl, and of oxygen have been recommended. Washing out the stomach has also been advised in cases where there is reason to suspect the presence of some gastric irritation. In addition to these internal means, various external applications may be found of value; these include mustard plasters round the diaphragm; pressure, galvanism, or faradism applied over the trunks of the phrenics just above the clavicles; blisters over the origin of the phrenics; the application of electricity to the stomach; and the use of baths.

In selecting any remedy or remedies we must always keep before us not only the probable cause of the symptom, but also the general state of the patient. For ordinary obstinate cases the use of bromides, of morphine hypodermically, or of belladonna, combined, at the same time, with an antispasmodic such as musk or oil of anethum, will often afford the desired relief. If the condition occur in chronic Bright or uræmia, pilocarpine or jaborandi may be of great value, while in hysterical cases valerianate of zinc internally, with the faradic current over the epigastrium (to act by suggestion), may bring the hiccough to an end. Whatever form of medicament be selected, it is essential at the same time to treat any co-existing malady, and not to rely simply on symptomatic treatment. In all bad cases the bowels must be attended to, and food administered in a light and easily digestible form, small meals being given at shorter intervals than usual to prevent overloading of the stomach.

With such an armamentarium to fall back upon as I have indicated above, we should be able to treat the most obstinate cases with success, thus earning the hearty gratitude of the patients who have been the unfortunate sufferers from this very distressing symptom.

